# Built and social environmental factors influencing healthy behaviours in older Chinese immigrants to Australia: a qualitative study

**DOI:** 10.1186/s12966-019-0885-3

**Published:** 2019-11-29

**Authors:** Ester Cerin, Andrea Nathan, Wing Ka Choi, Winsfred Ngan, Shiyuan Yin, Lukar Thornton, Anthony Barnett

**Affiliations:** 1 Mary MacKillop Institute for Health Research, Australian Catholic University, Melbourne, Australia; 2 School of Public Health, The University of Hong Kong, Hong Kong, Hong Kong SAR, China; 3 Baker Heart and Diabetes Institute, Melbourne, Australia; 4 Cancer Council Victoria, Melbourne, Australia; 5 School of Exercise and Nutrition Science, Deakin University, Geelong, Australia

**Keywords:** Older adults, Chinese immigrants, Neighbourhood environment, Physical activity, Walkability, Diet, Social contacts, Transport, Destinations

## Abstract

**Background:**

Neighbourhood environments influence older adults’ health and health-enhancing behaviours, such as physical activity, eating a healthy diet and socialising. However, little is known about the effects of the neighbourhood environment on the health of older immigrants, the number of which is rapidly increasing in developed countries. Using Nominal Group Technique (NGT) sessions, this study of older Chinese immigrants to urban Melbourne, Australia, examined built and social environmental facilitators of and barriers to regular engagement in physical activity, eating a healthy diet and regular contact with other people.

**Methods:**

Participants were recruited from four types of neighbourhoods stratified by walkability and proportion of Chinese dwellers. Twelve NGTs, four specific to each of physical activity, healthy diet and social contacts were conducted in Mandarin or Cantonese (91 participants). NGT responses from groups addressing the same questions were aggregated, similar items were combined, and scores combined across groups. Inductive thematic analysis was used to categorise answers into higher-order themes of factors associated with each behaviour.

**Results:**

For physical activity, 29 facilitators and 28 barriers were generated with the highest ranked facilitator and barrier being “proximity to destinations” and “poor/inadequate public transport”, respectively. For healthy diet, 25 facilitators and 25 barriers were generated, the highest ranked facilitator and barrier were “high food safety standards/regulations” and “lack of family/household members’ social support for a healthy diet”. The social contacts NGTs generated 23 facilitators and 22 barriers, with the highest ranked facilitator and barrier being “proximity to destinations and activities” and “poor public transport”, respectively.

**Discussion:**

Independent living arrangements and the accessibility of destinations of daily living (e.g., bilingual health services, libraries, places of worship and grocery stores / supermarkets), recreational facilities, affordable public transport, and community centres and activities for Chinese people are key elements for promoting regular engagement in physical activity, healthy eating and socialising in older Chinese immigrants. Governments should plan for the provision of this basic infrastructure of community facilities for older immigrants.

## Background

The neighbourhood environment is an important source of influences on older adults’ health [1–5] and health-enhancing behaviours, including participation in physical activity [6–8], eating a healthy diet [9, 10] and socialising [11, 12]. For example, older people living in neighbourhoods with better access to daily destinations and amenities have reported higher levels of participation in active transport [7], physical activity [6] and social activities [11, 13]. Neighbourhood environments are particularly relevant to older adults because they experience more limited mobility and are, therefore, more reliant on facilities near to where they live [4].

Studies of neighbourhood effects on health-enhancing behaviours have mainly focused on the general population of older adults living in specific areas, while there is a dearth of information on these issues in relation to older immigrants [14, 15]. Older immigrants are particularly susceptible to being impacted by their neighbourhood environment due to cultural differences and poor language proficiency after migration, which limit their mobility [16]. For this reason and the fact that the number of older immigrants to developed countries is rapidly increasing (e.g., increase of > 11 million from 1990 to 2017) [17], it is important to identify neighbourhood characteristics that influence health-enhancing behaviours of older immigrants in these regions. Oceania (specifically, Australia and New Zealand) is the developed region with the largest proportion of, and increase in, older immigrants in the world [17]. According to 2016 census data, Chinese immigrants are the largest non-English-speaking minority group in Australia [18], accounting for 2.7% of Australia’s total population and 1.6% of the total ageing (65+ year old) population [19]. As most immigrants to Australia are settling in capital cities, it is important to investigate characteristics of the neighbourhood environment that might impact on health-enhancing behaviours of older Chinese immigrants to urban Australia. This information can be used to help identify groups of immigrants that are particularly vulnerable to various health-related problems arising from dramatic environmental shifts (e.g., change in available foods, opportunities for physical activity and social contacts) and identify interventions that may mitigate these negative effects.

The aim of the present study was to identify built and social environmental facilitators of and barriers to regular engagement in physical activity, eating a healthy diet and regular contact with other people among older Chinese immigrants to urban Australia. We focused on Melbourne metropolitan area because Melbourne was the capital city with the largest population growth in 2016–17, with overseas migration accounting for 64.1% of the growth [18]. Given the dearth of information on the issues covered by the present study, a qualitative approach was implemented. Qualitative studies are key to informing intervention strategies that are effective and sustainable. This is because they enable the identification of high-priority problems and approaches that are feasible and acceptable to the target population. Qualitative research is particularly important for the planning and implementation of interventions for non-mainstream, hard-to-reach, isolated subgroups of the population (e.g., older Chinese immigrants to Western countries) because they are understudied and usually require approaches that differ from those that are effective in the mainstream population [20]. According to empirical evidence, intervention strategies tailored to minority groups that are based on findings from qualitative formative research are more effective than those that do not collect or utilise such information [21]. For the purpose of this study, a qualitative, structured, brainstorming technique (i.e., Nominal Group Technique, NGT) was adopted [22]. Compared to other qualitative techniques, such as focus groups, NGT sessions have been shown to produce a greater number of diverse ideas and facilitate balanced participation between group members [22].

## Methods

A qualitative study of older Chinese immigrants was conducted to identify built and social environmental facilitators and barriers to healthy behaviours. Ethics approval was granted by the Deakin University Human Ethics Advisory Group, Faculty of Health (reference number 161–2014).

### Participants

A purposive convenience sample of older Chinese immigrants was recruited from four types of neighbourhoods varying in walkability and proportion of Chinese dwellers. This approach ensured the recruitment of a heterogeneous sample of participants exposed to different levels of access to services, availability of Chinese ethnic foods and opportunities to socialise with residents of Chinese background.

Neighbourhoods in metropolitan Melbourne (defined as Statistical Areas Level 1, SA1; the smallest area of output for the Australian Census of Population and Housing) were identified and stratified as being high or low walkable using a transport-related walkability index in Geographic Information Systems (GIS) including information on dwelling density, street intersection density and land use mix. Low and high walkable neighbourhoods were those with a walkability index falling in the first and fourth quartiles, respectively. This strategy has been often employed in studies on the neighbourhood environment to maximise variation in types of environments [23–25]. Census data were used to classify neighbourhoods into those with a medium percentage of Chinese dwellers (5–10% of residents were Chinese) or high percentage of Chinese dwellers (> 10% of residents were Chinese). Thus, four categories of neighbourhoods were created: high walkable and high % Chinese (HW/HC); high walkable and medium % Chinese (HW/MC); low walkable and high % Chinese (LW/HC); and low walkable and medium % Chinese (LW/MC).

Participants were then recruited using a variety of approaches, such as advertisements in Chinese newspapers, delivering presentations at Chinese seniors’ groups and Chinese speaking churches, and posters in community libraries and medical centres. Presentations were delivered in Cantonese, Mandarin or English, depending on the audience preferences. All printed recruitment material was prepared in Traditional Chinese, Simplified Chinese and English. Potential participants contacted project staff speaking their preferred language (Cantonese, Mandarin or English) to assess eligibility status. Participants were eligible if they were ethnically Chinese and not born in Australia; aged 60 years and over; able to walk unassisted; able to speak and read Chinese (Cantonese/Mandarin) and/or English; able to attend a data collection session lasting up to 1.5 h; did not suffer from a diagnosed medical condition that affects cognitive functioning (e.g., dementia or major depression); and lived in pre-selected neighbourhoods. Participants were recruited between October and November 2014. If eligible, then written consent was provided.

### Data collection

Participants completed a 15 item Demographic and Health questionnaire, which was returned along with a signed consent form. NGT sessions were held from October 2014 to March 2015. NGT is a well-established structured, multistep, ‘brainstorming’ technique used to generate and prioritise responses from a group of participants to a carefully articulated question intended to address specific information needs [22]. Overall 12 NGT sessions were held, four sessions specific to each of three healthy behaviours: physical activity (defined as ‘any type of activity that uses your muscles and uses more energy than just resting’), healthy diet (defined as ‘eating a wide variety of different foods including plenty of fruits and vegetables, nuts, whole grains, beans and peas, lean meats, fish and chicken; and eating foods that are fatty, salty or sweet sparingly’), and social contacts (defined as ‘regular pleasant contacts with other people in the place where you live, here in Australia, such as friends, family members, colleagues, neighbours, acquaintances or people who work in your local area (e.g., shop assistant)). The NGT sessions involved a total of 91 participants, ranging from five to 13 participants per session. Each participant was randomly assigned to and took part in only one NGT session on a particular healthy behaviour. NGT sessions were held at Deakin University or at a local community centre and conducted in either Mandarin or Cantonese (the participants’ preferred languages). The NGT sessions lasted 60–90 min.

Three research assistants were trained to facilitate sessions. All sessions followed the same structure and procedures adopted in previous NGT studies seeking to identify built and social environmental factors associated with healthy behaviours [24, 26, 27]. Participants were told that the purpose of the NGT was to provide ideas on what they thought could be changed in living environments to create better and healthier places for elderly Chinese to live in. First, participants were asked to “list things about places where Chinese elderly live that make it easy to be physically active, eat healthy, or have contacts with people”. Participants were told that it could be about people in those places, buildings, facilities, services or infrastructure. They then wrote down ideas on a piece of paper without discussing them with other participants. Once the response generation process was completed, the facilitator asked participants to nominate the responses written on their own worksheet using a round-robin approach until all ideas were exhausted. All responses were written on a whiteboard for the participants to read. Generated items were reviewed and clarified if ambiguous or combined if equivalent. Once finalised, each participant anonymously selected and wrote down the three most relevant items on a blank card and ranked them by importance using scores from 1 to 3 (3 denoting the most important item). In the second part of the NGT session participants were asked to “list aspects of places where Chinese elderly live that make it difficult to be physically activity, eat healthy, or have contacts with people”. The same round-robin listing and voting steps then occurred. For the third and final part, participants were asked “if they could change one thing about the place they live in that would help them exercise more, have a healthy diet, or have contacts with people, what would it be?”. No round-robin listing or voting occurred for this third part. The main purpose of the third part of the data collection was to identify potential interventions that would have local relevance and compare those with the more general list of facilitators / barriers to health-enhancing behaviours identified by the same participants.

### Analysis

Differences in socio-demographic and health-related characteristics between behaviour-specific NGT groups were tested using chi-squared tests. All NGT responses were entered in a database. The responses from NGT groups addressing the same questions about a specific healthy behaviour were aggregated. Specifically, items that were very similar were first combined to create a new list of distinctive (non-overlapping) items. The total score for each distinctive item was computed by summing the scores it received from each participant (possible individual participant’s scores ranged from 0 to 3). In the present study, the minimum score that an item could receive was 0. This could occur if no participants deemed the specific item to be one of the three most relevant generated items. The maximal score that an item could receive was equal to the total number of participants taking part in one of the four behaviour-specific NGTs multiplied by 3 (e.g., a total of 32 participants participating in physical-activity-focused NGTs could yield a maximum item score of 96). This could occur if all participants deemed the specific item to be the most relevant in the list and gave it a score of 3. Inductive thematic analysis was used to identify and further categorise items into higher-order themes of factors associated with each behaviour (e.g., the environmental physical activity facilitators “park nearby” and “cycling track nearby” were categorised under the theme “proximity to destinations”). Three investigators reviewed these aggregations, discussed differences in opinions and resolved disagreements. Scores for specific themes within each healthy behaviour (e.g., proximity to destinations as facilitators of engagement in physical activity) were then computed by aggregating (i.e., summing up) the scores on the relevant items.

## Results

### Participant characteristics

Overall, 91 older Chinese immigrants participated in the 12 groups held: four NGTs on physical activity, healthy diet, and social contacts, respectively. Socio-demographic and health-related characteristics are presented in Table 1 stratified by NGT healthy behaviour. Participant ages ranged from 60 to 85 years (M = 71.13, SD = 5.91). Across all participants, duration of residency in Australia ranged from 1 to 58 years (M = 12.12, SD = 12.41). Most participants lived with their adult children. The distributions of participants by neighbourhood walkability (*p* = 0.640), neighbourhood-level percentage of Chinese dwellers (*p* = 0.775), sex (*p* = 0.851), age groups (*p* = 0.220), ethnicity (*p* = 0.739), language spoken at home (*p* = 0.483), residency in Australia (*p* = 0.733), living arrangements (*p* = 0.904), self-reported health (*p* = 0.627) and number of chronic conditions (*p* = 0.317) did not differ across the three behaviour-specific NGTs.

**Table 1:** Participant socio-demographic and health-related characteristics by NGT topic

		Physical activity (*n* = 37)		Healthy diet (*n* = 27)		Social contacts (n = 27)		Total (*n* = 91)	
		n	%	n	%	n	%	n	%
Neighbourhood walkability	High	18	48.6	16	59.3	13	48.1	47	51.7
	Low	19	51.4	11	40.7	14	51.9	44	48.3
Neighbourhood % Chinese	Medium	16	43.2	14	51.9	12	44.4	42	46.1
	High	21	56.8	13	48.1	15	55.6	49	53.9
Sex	Male	14	37.8	9	33.3	11	40.7	34	37.4
	Female	23	62.2	18	66.7	16	59.3	57	62.6
Age group	60–64 years	11	29.7	5	18.5	1	3.7	17	18.7
	65–69 years	6	16.2	8	29.6	8	29.6	22	24.2
	70–74 years	6	16.2	6	22.2	10	37.0	22	24.2
	75–79 years	11	29.7	6	22.2	6	22.2	23	25.3
	80+ years	3	8.1	2	7.4	2	7.4	7	7.7
Place of origin	Shanghai province	10	27.0	8	29.6	4	14.8	22	24.2
	Guangdong province	6	16.2	3	11.1	6	22.2	15	16.5
	Beijing province	4	10.8	3	11.1	4	14.8	11	12.1
	Other provinces^a^	12	32.4	6	22.2	6	22.2	24	26.4
	Hong Kong SAR	4	10.8	3	11.1	6	22.2	13	14.3
	Taiwan	0	0.0	1	3.7	0	0.0	1	1.1
	Other^b^	1	2.7	3	11.1	1	3.7	5	5.5
Language spoken at home	Mandarin only	20	54.1	14	51.9	13	48.1	47	51.6
	Cantonese only	7	18.9	8	29.6	11	40.7	26	28.6
	Other^c^	10	27.0	5	18.5	3	11.1	18	19.8
Residency in Australia	< 5 years	20	54.1	10	37.0	11	40.7	41	45.1
	5–14 years	6	16.2	6	22.2	6	22.2	18	19.8
	15–24 years	5	13.5	7	25.9	4	14.8	16	17.6
	25+ years	6	16.2	4	14.8	6	22.2	16	17.6
Living arrangements	Living alone	2	5.4	2	7.4	3	11.1	7	7.7
	Living with others but not adult children	14	37.8	10	37.0	8	29.6	32	35.2
	Living with adult children	21	56.8	15	55.6	16	59.3	52	57.1
Self-rated health	Poor	1	2.7	0	0.0	3	11.1	4	4.4
	Fair	15	40.5	11	40.7	10	37.0	36	39.6
	Good	12	32.4	12	44.4	8	29.6	32	35.2
	Very good	8	21.6	4	14.8	6	22.2	18	19.8
	Excellent	1	2.7	0	0.0	0	0.0	1	1.1
Number of chronic health conditions	None	9	24.3	10	37.0	4	14.8	23	25.3
	One	11	29.7	8	29.6	7	25.9	26	28.6
	Two or more	17	45.9	9	33.3	16	59.3	42	46.2

^a^Other includes Chongqing, Sichuang, Anhui, Fujian, Jiangsu, Shandong, Zhejiang, Heilongjiang, Liaoning, Shanxi, Tianjin provinces. ^b^Other includes Malaysia, Vietnam. ^c^Other includes English, Chiu Chau dialect, Shang Hai dialect, Southern Fujian dialect, Vietnamese, Taiwanese or any combination of these with Mandarin and/or Cantonese

### Environmental factors related to physical activity

A total of 57 responses (29 facilitators and 28 barriers) were generated. The aggregated responses ranked as important across all four NGT groups are presented in Table 2, while more detailed sub-categories of responses, including those that no participant considered among the three most important, can be found in Additional file 1: Table S1. The highest ranked facilitator of being physically active was proximity to destinations, endorsed by three out of four NGT groups (including both groups of participants living in low walkable areas). Specifically, participants reported that living close to parks, recreational facilities, and shopping centres made it easy to be physically active. Having access to destinations for physical activity (without specifying how far away from home they were located) was the next highest ranked environmental facilitator. Next was the importance of having access to organised social groups and structured and diverse activities to participate in, which was the only facilitator endorsed by all four group types. Other responses endorsed by more than one NGT group included aspects of the home environment supporting physical activity (particularly important to participants from low walkable areas); social support for physical activity (endorsed by two out of four group types, both of which were living in high walkable areas); and pet ownership.

**Table 2:** Built and social environmental facilitators and barriers to engagement in physical activity

Categories of responses generated during NGT sessions	Total votes (across groups)^a^	Group types endorsing response^b^
Facilitators		
Proximity to destinations	63	LW/HC, LW/MC, HW/HC
Easy access to destinations for physical activity (regardless of distance)	34	HW/MC, LW/HC
Access to social group/activities	28	HW/MC, LW/HC, LW/MC, HW/HC
Information available on health and community events, initiatives or services in Chinese	18	HW/MC
Home environment providing opportunities for physical activity	17	LW/HC, LW/MC, HW/HC
Social support for physical activity	14	HW/MC, HW/HC
Enjoying household physical activity [characteristic describing Chinese older residents in the community]	14	HW/HC
Opportunities to facilitate integration in the community	13	HW/MC
Quality public transport	8	HW/MC
Pet ownership	5	LW/MC, HW/HC
Safety from traffic	5	LW/MC
Caring responsibilities	2	HW/HC
Living near other Chinese elders	1	LW/MC
Barriers		
Poor/inadequate public transport	40	HW/MC, LW/HC, LW/MC, HW/HC
Language barriers	36	HW/MC, LW/HC
Lack of destinations/facilities supporting physical activity	34	LW/HC, LW/MC
Limited social group/activities for Chinese people	29	HW/MC, HW/HC
Health-related and socio-economic factors [characteristics describing Chinese older residents in the community]	28	HW/MC, HW/HC
Lack of information on community activities	23	HW/MC, LW/HC
Home environment not conducive to physical activity	20	LW/HC, LW/MC
Lack of social support for physical activity	9	LW/HC
Lack of public housing supporting independence	3	HW/MC

^a^Maximum of 6 votes per participant (3–2-1 scoring). ^b^HW = High Walkability; LW = Low Walkability; MC = Medium % Chinese; HC = High % Chinese

The highest ranked barrier to being physically active was poor/inadequate public transport, which was the only response endorsed by all four NGT groups. Other environmental barriers to physical activity endorsed by more than one NGT group included: language barriers; lack of destinations supporting physical activities (important to residents of low walkable areas); having limited social groups/activities to join (endorsed by the two high walkable NGT groups); individual health-related and socio-economic factors (endorsed by participants living in high walkable areas); lack of information about community activities; and aspects of the home environment not conducive to physical activity (endorsed by the two groups living in low walkable areas).

When asked “if you could change one thing about your place you live in that would help you engage in more physical activity, what would it be?”, the most popular responses were (in order): access to and improvement of Chinese community centres with facilities for recreational physical activity; improving living arrangements; proximity of destinations for recreational physical activity; and reducing language barriers. Each of these items was raised by participants from at least two NGT groups (Table 3 and Additional file 1: Table S2).

**Table 3:** One thing that Chinese older immigrants would change in their own community to help them be more physically active, eat a healthy diet and have contacts with people

Categories of responses	Total votes (across groups)	Group types endorsing response^a^
Physical activity		
Access to and improvement of Chinese elderly community centres with facilities for recreational physical activity	13	LW/HC, LW/MC, HW/HC, HW/MC
Improving living arrangements	12	HW/MC, LW/HC
Proximity to destinations for recreational physical activity	5	HW/MC, LW/MC
Reducing language barriers	4	HW/MC, LW/HC
Improvements in the neighbourhood environment	1	HW/MC
Reducing financial barriers	1	HW/HC
Opportunities to facilitate integration	1	HW/MC
Healthy diet		
Better provision of educational information on healthy eating in the community	7	LW/HC, LW/MC, HW/MC
Better access to grocery stores and/or fresh produce	7	HW/MC, LW/HC, HW/HC
Improve access to and/or management of Chinese grocery stores	4	LW/MC, HW/HC
Improved public transport	4	HW/MC, LW/MC
Reduce unhealthy food options in food outlets	2	LW/HC, HW/HC
Interventions aimed at improving diet-related habits	2	HW/HC
Financial independence	1	HW/MC
Social contacts		
Improving public transport	7	HW/MC, LW/HC, LW/MC
Improving access to places for social/group activities for Chinese elderly	7	LW/HC, HW/MC
Increasing the number of activities in the community for Chinese elderly	3	HW/HC
Provision of opportunities for the improvement of English language skills	2	HW/MC, LW/MC
Provision of services in Chinese	2	LW/MC
Living near other Chinese people	2	LW/MC, HW/MC
Promoting neighbourliness	1	LW/HC
Housing design to facilitate social contacts	1	LW/MC
Instrumental support for mobility	1	LW/MC
Improved access to telecommunication	1	LW/MC

^b^HW = High Walkability; LW = Low Walkability; MC = Medium % Chinese; HC = High % Chinese

### Environmental factors related to a healthy diet

Overall, 25 facilitators and 25 barriers (50 in total) were generated. Table 4 outlines the aggregated categories of facilitators and barriers to following a healthy diet across all four NGTs, while Additional file 1: Table S3 also reports sub-categories of barriers and facilitators. The highest ranked facilitator, which was endorsed by all four NGT groups, was having high food safety standards/regulations. This was followed by the provision of educational information on healthy eating through various outlets (Chinese newspapers, community talks), which was endorsed by three out of four NGT groups. Other frequently-endorsed facilitators to eating a healthy diet included availability of healthy foods in grocery stores and having family/household social support for a healthy diet.

**Table 4:** Built and social environmental facilitators and barriers to a healthy diet

Categories of responses generated during NGT sessions	Total votes (across groups)^a^	Group types endorsing response^b^
Facilitators^c^		
High food safety standards/regulations	59	HW/MC, LW/HC, LW/MC, HW/HC
Providing educational information on healthy eating in the community	44	HW/MC, LW/HC, LW/MC
Family/household members social support for a healthy diet	22	LW/MC, HW/HC
Availability of healthy foods in grocery stores	20	HW/MC, HW/HC, LW/HC
Food nutritional labelling in Chinese	13	LW/MC, LW/HC, HW/HC
Preference for a healthy diet and healthy cooking practices [characteristic describing Chinese older residents in the community]	9	LW/MC, HW/HC
Proximity to Chinese grocery store	7	LW/MC
Barriers		
Lack of family/household members social support for a healthy diet	54	HW/MC, LW/HC, LW/MC, HW/HC
Financial barriers to purchasing healthy foods	26	HW/MC, HW/HC
High availability or prevalence of unhealthy food options available in food outlets	23	HW/MC, HW/HC
Cultural preference for an unhealthy diet and unhealthy cooking practices [characteristic describing Chinese older residents in the community]	19	LW/MC, LW/HC, HW/HC
Distance to grocery store	14	HW/MC, LW/MC
Limited availability of fresh vegetables	9	HW/HC
Poor adherence to food safety standards/regulations	6	HW/HC
Unable to grow fruit and vegetables at home	4	HW/MC
Language barriers to reading food nutritional labels	4	LW/MC
Poor public transport (low frequency)	2	LW/MC
Misleading or inaccurate educational information on healthy eating	1	LW/MC

^a^Maximum of 6 votes per participant (3–2-1 scoring). ^b^HW = High Walkability; LW = Low Walkability; MC = Medium % Chinese; HC = High % Chinese. ^c^Additional votes = 12 additional votes for ‘Facilitators’ due to scored items listing two factors being counted twice under different response categories

Endorsed by all NGT groups, the highest ranked barrier to healthy eating was to do with a lack of family/household support for healthy food options. Other barriers endorsed by more than one NGT group were: financial barriers to purchasing healthy foods (endorsed by residents of high walkable areas); high availability or prevalence of unhealthy food options available in food outlets (endorsed by residents of high walkable areas); distance to grocery stores (endorsed by respondents living in areas with medium percentage of Chinese residents); and cultural preferences for an unhealthy diet and unhealthy cooking practices (endorsed by both NGT groups living in high walkable areas).

When asked to identify one aspect to change that would help to follow a healthy diet, the most popular responses were having better provision of educational information on healthy eating and better access to grocery stores and/or fresh produce (mentioned by three NGT groups) (Table 3 and Additional file 1: Table S4). Other aspects mentioned by more than one NGT group were improved access to and/or management of Chinese grocery stores, improved public transport; and reduction of unhealthy food options in food outlets.

### Environmental factors related to social contacts

The total number of responses generated was 45 (23 facilitators and 22 barriers). Table 5 presents the aggregated categories of facilitators and barriers to having contact with people across all four NGTs. Sub-categories of facilitators and barriers are detailed in Additional file 1: Table S5.

**Table 5:** Built and social environmental facilitators and barriers to social contacts

Categories of responses generated during NGT sessions	Total votes (across groups)^a^	Group types endorsing response^b^
Facilitators^c^		
Proximity to destinations and activities	72	LW/HC, LW/MC, HW/HC, HW/MC
Availability of community services and media in Chinese	24	HW/MC, HW/HC, LW/MC
Access to destinations and activities	19	HW/MC, LW/MC, HW/HC
Opportunities to learn English	19	HW/MC, LW/MC
Good public transport	18	LW/MC, HW/HC
Living near or with Chinese people	10	LW/HC, LW/MC
Unlimited access to telecommunication	2	LW/MC
Barriers		
Poor public transport	66	HW/MC, LW/HC, LW/MC, HW/HC
Language barriers	49	HW/MC, LW/HC, LW/MC, HW/HC
Limited or poor access to destinations and social group/activities for Chinese people (or residents)	41	HW/HC, HW/MC, LW/MC
Living separately from other Chinese people (public housing arrangements)	5	LW/MC
Cultural differences limiting integration	4	HW/HC
Health-related and personality factors [characteristics describing Chinese older residents in the community]	3	HW/MC

^a^Maximum of 6 votes per participant (3–2-1 scoring). ^b^HW = High Walkability; LW = Low Walkability; MC = Medium % Chinese; HC = High % Chinese. ^c^Additional votes = 1 additional votes for ‘Facilitators’ and 6 for ‘Barriers’ due to scored items listing two factors being counted twice under different response categories

The highest ranked facilitator to social contact was proximity to destinations and activities, endorsed by all NGT groups. Specific destinations mentioned by participants included shops (especially Chinese stores), library, recreational facilities, GPs, restaurants, and community centres. Endorsed by both NGT groups living in high walkable areas, the next highest facilitator to social contact was the availability of community services and media in Chinese. This was followed by the following facilitator categories endorsed by at least two NGT groups: opportunities to learn English (endorsed by both groups living in areas with a medium percentage of Chinese residents); access to destinations and activities (endorsed by three of four NGT groups); good public transport; and living near or with Chinese people (endorsed by both NGT groups living in low walkable communities).

Two barriers to maintaining contact with people were ranked highly and endorsed by all four NGT groups. These were poor public transport, particularly its inconvenience and infrequency, and language barriers. Limited or poor access to destinations and social group/activities for Chinese people was also a highly ranked barrier to social contacts, which was endorsed by three out of four NGT groups.

The most popular changes to make in the community that would help older Chinese to have contacts with people were: improving access to places for social/group activities for Chinese elderly; improving public transport; and increasing the number of activities in the communities for Chinese elderly (Table 3 and Additional file 1: Table S6). The first two types of changes were endorsed by at least two NGT groups.

## Discussion

Although population ageing, urbanisation and international migration to developed countries are three main global demographic trends [28], there is a general dearth of information on how the urban environment of a host country impacts on the well-being of older immigrants. This qualitative study aimed to address this knowledge gap by identifying built and social environmental facilitators and barriers to three health-enhancing behaviours in older Chinese immigrants to Melbourne, Australia’s second largest city.

### Facilitators and barriers to engagement in physical activity

Regular engagement in physical activity is associated with a plethora of health benefits including risk reduction of premature death [29] and major chronic diseases [30]. Mid- and older-aged East Asian immigrants (mainly Chinese) to Australia have been found to be less physically active than non-immigrants [31], although Chinese older adults residing in their country of origin exhibit much higher levels of physical activity than older Australians [32–35]. These differences in activity levels have been in part attributed to better accessibility to destinations and public transport in Chinese than Australian cities [32, 36]. The results from the present study reiterate the importance of proximity and access to destinations as key facilitators to engagement in physical activity for older Chinese immigrants residing in both low- and high-walkable neighbourhoods relative to the local context of Melbourne, Australia. Also, the lack of destinations was identified as a major barrier to physical activity only by those living in low walkable areas typified by low levels of mixed-use buildings.

Types of destinations considered to be particularly important were parks, walking tracks, shops, recreational centres and community centres. Except for community centres, all these types of destinations have been associated with higher levels of physical activity among older adults in general [6–8]. Community centres appear to be particularly important to older immigrants. In this study, access to Chinese elderly community centres (e.g., Chinese seniors club) with facilities for physical activity was the most frequently endorsed single change in the community that participants thought would help them be more physically active. Previous studies have indicated that ethnic-specific community organisations are important sources of activities for older immigrants that, apart from offering affordable opportunities to engage in a particular activity (e.g., exercise), help them overcome social isolation and problems associated with language barriers by providing access to people of the same ethnic minority [37–39]. In connection with this issue, the present study found that participants from all four types of communities reported access to group (physical) activities as a major facilitator, and lack of group activities as a major barrier, to being physically active. It appears that the ability to link physical activities with local Chinese-speaking social networks is an important consideration for the promotion of physical activity in Chinese older immigrants [37].

As noted above, mirroring findings from other studies on older Chinese immigrants [38], this study suggests that factors related to language barriers and ways to mitigate them were deemed to influence the ability to engage in physical activity. Apart from access to group activities involving other Chinese-speaking residents, language-related facilitators/barriers identified in this study included the provision/lack of information on activities in Chinese, networking opportunities for English and Chinese older adults, and the (in) ability to communicate in English. According to Ip and colleagues [38], the lack of English language proficiency and the difficulties in accessing language support services, limit Chinese older adults’ mobility and, hence, ability to find and engage in community activities, including leisure-time physical activity appropriate for their age. Furthermore, inadequate and infrequent public transport services were the most frequently reported mobility-related barriers to engagement in physical activity by all NGT groups. Given that the majority of older Chinese immigrants do not drive [37, 38], their participation in activities outside their immediate neighbourhood is dependent on their family members (e.g., children driving them to/from destinations) and/or access to public transport. The latter emerged as a particularly important factor in the present study, likely due to Chinese older immigrants preferring not to rely on their children for transportation because they see them as being ‘always busy at work’ [37, 38].

Household environment characteristics (e.g., having a garden or exercise equipment at home) and individual-level characteristics of Chinese older immigrants (e.g., enjoying household activities, health problems, financial hardship, lack of time and pet ownership) were less frequently reported as factors impacting on engagement in physical activity. Most of these factors have previously been identified as physical activity barriers or facilitators in general populations of adults and older adults [40–42]. Among these factors, financial problems are particularly relevant to older immigrants who tend to be financially dependent on their children because they are less likely to accumulate adequate resources for independent living and often have reduced government pensions and benefits due to their immigrant status [37, 43]. As a result of these financial constraints, their choice of activities in the community are limited.

Linked to financial independence are living arrangements. Older Chinese immigrants often live with their adult children and children-in-law due to economic constraints, language barriers and cultural reasons [37, 38, 44, 45]. However, most of them express a desire for independent living arrangements, i.e., for living in a household separate from their children [37, 45]. Typically, the main reasons behind this desire are poor-quality relationships at home [38, 45, 46], seeking more autonomy in lifestyle and ability to fulfil one’s own needs [37, 38]. This explains why, in the present study, the provision of independent living arrangements was considered one of the main single changes that would help older Chinese immigrants be more physically active. Specifically, participants maintained that independent living would provide them the freedom and time to engage in leisure-time physical activity. Also, they indicated that public housing for Chinese elderly would facilitate participation in group activities and improve their mood and desire to participate in such activities.

### Facilitators and barriers to a healthy diet

While mid-aged and older East Asian (including Chinese) immigrants to Australia have been found to report lower fruit and vegetable intake than their Australian counterparts [31], Osypuk and colleagues [47] reported that Chinese who lived in immigrant enclaves had healthier diets that individuals who did not. Further, the diets of ethnic minorities who migrate to Western countries are usually worse than when living in their country of origin [48]. These findings suggest that healthy eating among older Chinese immigrants may be determined by the physical and social attributes of their home and neighbourhood environments. However, these issues remain understudied.

In this study, having high food safety standards was by far the most frequently endorsed facilitator to healthy eating. Food safety in China has been and is remaining a major concern [49]. In this regard, Alcorn and Ouyang [50] reported that Chinese residents considered food safety the second greatest risk they faced in daily life (after earthquakes). It is, thus, perhaps not surprising that older Chinese immigrants, most of whom had migrated to Australia from China in recent times (< 5 years), would consider high food safety standards and regulations an essential factor for healthy eating. However, they did not identify low food safety standards as barriers to healthy eating, nor did they select improvements in food safety standards as the single thing they would change in their host community to help them follow a healthier diet. These patterns of findings are likely reflective of their confidence in Australian food safety standards which are higher than those in China [51].

In this study, the provision of educational information on healthy eating was the second most frequently endorsed facilitator to healthy eating and one of the two most popular single changes older Chinese immigrants thought would help them adopt or sustain a healthy diet. Participants emphasized the need for information in Chinese that would help them address language barriers. They noted the utility of having information on healthy eating published in the local Chinese newspapers and/or delivered through talks and workshops (in Chinese) organised by local community groups and/or health services.

Improvements in some eating habits (e.g., decreased use of deep-frying, increased fruit and vegetable intake) as a result of improved nutritional knowledge have been previously observed in Chinese immigrants to Western countries [52–54]. These findings are in line with research on mainstream populations. In fact, nutritional knowledge has been consistently identified as one of the most important psychosocial determinants of a healthy diet. For example, in their systematic review, Guillaumie and colleagues [55] noted that over half of the studies that examined the effects of knowledge on fruit and/or vegetable intake reported positive effects. Also, a recent study found that knowledge was a strong predictor of vegetable intake in East Asians [56].

Two strong psychosocial determinants of healthy eating in mainstream populations that emerged as key facilitators/barriers in this study are social influences [55, 56] and eating/cooking habits [55]. As noted earlier, a large proportion of older Chinese immigrants live with, and depend on the financial support of, their adult children [37, 43]. These constrained living arrangements limit their freedom to choose the foods they eat. In this study, living with family members (mainly children) who prefer unhealthy fast foods was by far the most prevalent perceived barrier to healthy eating. It has been suggested that younger Chinese immigrants tend to more easily adopt a Western diet, while older Chinese immigrants prefer a healthier traditional diet [54]. However, their ability to follow their dietary preferences depends on their financial independence and status in the household [37]. In this regard, it is notable that, in the present study, financial constraints were the second most highly ranked perceived barrier to healthy eating, further highlighting the disadvantaged socio-economic position of older Chinese immigrants. In line with these findings are those of mainstream studies that have identified healthy food affordability as a consistent determinant of healthy food choices and purchasing behaviours being particularly important to low-income older adults [57, 58].

This study identified comparative availability of healthy and unhealthy foods in the community and grocery stores / supermarkets as an important factor impacting on healthy eating. Specifically, the availability of fresh produce and affordable fruit and vegetables in supermarkets were considered facilitators of healthy eating by participants living in different types of neighbourhoods. On the other hand, the availability of unhealthy foods in supermarkets and restaurants, and the limited choice of fresh produce in grocery stores were identified as barriers among those living in high walkable areas. The same group of participants indicated that an increase in amounts and varieties of fruit and vegetables in grocery stores and supermarkets would help them follow a healthy diet. Residents of high walkable areas usually have relatively good access to supermarkets and shops [7]. Thus, for them, availability of healthy foods rather than access to outlets that sell such foods may be a more salient determinant of healthy eating. Previous studies have noted that the comparative availability of healthy and unhealthy options in food outlets played a key role in residents’ dietary patterns [58]. This was particularly the case among low-income [59] and ethnic minority groups [60], with the latter reporting difficulties in obtaining traditional ethnic-specific fresh produce.

Similarly to that observed in mainstream populations [58], older Chinese immigrants indicated that accessibility of healthy foods in the community – namely, distance to grocery stores - influenced their diet. Having access to grocery stores (Chinese or generic), markets and good public transport to/from grocery stores were among the single changes they would introduce in the local community to help them eat healthily. Lack of transportation to access grocery stores and long distances to grocery stores were aspects of the neighbourhood environment that emerged as important barriers to healthy eating in other studies on socially disadvantaged older adults [57] and other age groups [58].

### Facilitators and barriers to social contacts

Socially isolated individuals are at increased risk of dying prematurely [61] and not recovering from life threatening events [62]. Social isolation and feelings of loneliness are also associated with depression [63], mental and cognitive health decline [64], and poorer sleep [65] and quality of life [46]. Older Chinese immigrants to Western countries are particularly vulnerable to social isolation because of their older age, relocation to an unfamiliar environment, insufficient language skills to build new networks, lack of information and access to services, and cultural barriers [66, 67]. They frequently report insufficient quantity of social relationships outside their household [45] which, in conjunction with language and cultural barriers, leads to them feeling lonely [46]. It is, thus, important to identify ways to promote and enhance social contacts in this population.

Three main sources of influences on the older Chinese immigrants’ ability to socialise emerged in the present study. These were access and availability of appropriate places and activities to meet people, language barriers and transportation. Proximity and access to destinations and activities were the facilitators most frequently endorsed by all NGT groups. Also, improvements in these community characteristics were identified as key interventions that would help older Chinese immigrants have contact with other people. In particular, participants emphasised the importance of having access to activities and destinations for Chinese residents, including Chinese community centres, senior clubs, libraries, churches and health services. Proximity to shops and restaurants was also found to be a positive contributor to social activities. Previous studies on older Chinese immigrants found that Chinese community centres and services were vital sources of social networks [38, 39, 45, 68]. Makwarimba and colleagues [68] noted that older immigrants want to have their own ethnic senior centres where they can meet people who speak their own language, cook their ethnic food and access service information through dissemination activities. This is a particularly important issue for older Chinese immigrants co-residing with their adult children, lacking private space for socialising at home, and experiencing strained relationships with their younger household members [37, 38, 46] that result in lower levels of psychological well-being [69].

Having access to destinations and activities for Chinese people was one of the ways in which participants could address their lack of proficiency in the English language as a barrier to socialising. Other ways were living near other Chinese people and having opportunities to learn English. In this regard, it was suggested that the local community and government should provide English language classes for older immigrants and public housing for Chinese people. In an earlier study of older Chinese immigrants to Australia, Ip and colleagues [38] found that one of the consequences of poor English language skills was that older immigrants did not have the confidence to venture out on their own to visit their friends or attend social activities, which reinforced their social isolation.

Finally, in this study, access to good public transport was identified as a facilitator and poor public transport as the most important barrier to having social contacts. Participants living in communities differing in level of walkability and prevalence of Chinese residents all perceived public transport inconvenience, unaffordability and unintelligibility (e.g., public transport stop signs and bus numbers being unclear) as a major barrier. Similar findings were observed in other studies on older Chinese in Australia [38] and Canada [45].

### Study limitations

This study has several limitations. Firstly, NGT procedures do not allow participants to elaborate on the reasons for selecting specific facilitators or barriers to health-enhancing behaviours. NGTs were conducted with a non-probabilistic, purposively selected sample of older Chinese immigrants to one Australian city. Hence, the findings are not generalizable to all older Chinese immigrant groups in Australia and in other countries. The characteristics of the built and social environment in other Australian cities and countries are likely to differ [70, 71] and impact on the perceived factors influencing participation in physical and social activities as well as the ability to follow a healthy diet. Also, the findings may not apply to other ethnic groups. Future work should include larger and more representative samples of older Chinese from diverse geographical locations.

### Practical implications

Despite the limitations of this study, the findings have useful practical implications. The household and community environments are particularly important to older Chinese immigrants because they determine their level of independence and access to healthy foods, affordable transportation, opportunities for physical and social activities, and social networks that provide information and support. The provision of public housing in walkable neighbourhoods with access to destinations for daily living, recreational facilities and affordable public transport would enable older Chinese immigrants to live independently from their adult children and, as a result, follow a healthier diet and have more time for engaging in physical and social activities with other older Chinese residents. Destinations for daily living that should be provided within walking distance from residential buildings are Chinese or bilingual health services, libraries, places of worship, and grocery stores / supermarkets selling affordable ethnic-specific fresh produce. It would be particularly important to establish a dedicated community centre for older Chinese that would provide a place for physical and social activities and be a dissemination point for information about community events, health and government policies. Local communities should also offer multi-purpose programs, such as English classes that promote physical activity and healthy eating, which have been shown to be effective in minority groups [72].

The present study confirms the utility of qualitative research in identifying problems (e.g., isolation) and solutions (e.g., English classes that promote healthy eating) related to minority groups’ healthy behaviours that do not typically apply, or do not apply to the same extent, to the mainstream population. The findings from this and future similar qualitative investigations can be used to inform the planning and implementation of tailored interventions aimed at promoting a physically and socially active lifestyle and healthy eating in older Chinese immigrants to Western countries. Within the context of Melbourne, the current study suggests that effective and sustainable interventions would need to focus on environmental changes that improve the independence and decrease the level of isolation of older Chinese immigrants. The types of interventions identified by study participants (e.g., the provision of public housing, affordable public transport and ethnic-specific fresh produce) would require the coordinated engagement of state and local transportation, urban planning, health and human services departments in consultation with representatives of the community. In order to respond to community needs, policymakers and practitioners ought to be informed of the existence of these needs and possible ways to meet them. The provision of research summaries from this and similar qualitative studies that also contain policy recommendations may facilitate research translation and community-level action [73].

## Conclusions

This study examined physical and social environmental facilitators and barriers to physical activity, healthy eating and social contacts in older Chinese immigrants to Melbourne, Australia. In line with previous investigations conducted in other Western, English-speaking geographical locations, the present study suggests that independent living arrangements and the accessibility of destinations of daily living, recreational facilities, affordable public transport and community centres and activities for Chinese people are key elements for promoting a healthy lifestyle and well-being in older Chinese immigrants. As immigration to developed countries is likely to continue in the future, governments should plan for the provision of this basic infrastructure of community facilities for older immigrants.

## Additional file

**Additional file 1: Table S1.** Built and social environmental facilitators and barriers to physical activity. **Table S2.** One thing that Chinese older immigrants would change in their own community to help them be more physically active. **Table S3.** Built and social environmental facilitators and barriers to eating a healthy diet. **Table S4.** One thing that Chinese older immigrants would change in their own community to help them follow a healthy diet. **Table S5.** Built and social environmental facilitators and barriers to social contacts. **Table S6.** One thing that Chinese older immigrants would change in their own community to help them have contacts with people.

## Data Availability

Available on request.

## Abbreviations

HC: High neighbourhood proportion of Chinese dwellers
HW: High walkable
LC: Low neighbourhood proportion of Chinese dwellers
LW: Low walkable
NGT: Nominal Group Technique
