# Supplementary material for: Built and social environmental factors influencing healthy behaviours in older Chinese immigrants to Australia: a qualitative study
Source: Int J Behav Nutr Phys Act. 2019 Nov 29;16:116. doi: 10.1186/s12966-019-0885-3 (PMC6883540; doi:10.1186/s12966-019-0885-3)
Supplement: Supplementary file 1 — Additional file 1: Table S1. Built and social environmental facilitators and barriers to physical activity. Table S2. One thing that Chinese older immigrants would change in their own community to help them be more physically active. Table S3. Built and social environmental facilitators and barriers to eating a healthy diet. Table S4. One thing that Chinese older immigrants would change in their own community to help them follow a healthy diet. Table S5. Built and social environmental facilitators and barriers to social contacts. Table S6. One thing that Chinese older immigrants would change in their own community to help them have contacts with people. [file 12966_2019_885_MOESM1_ESM.docx]

**Supplementary Table 1: Built and social environmental facilitators and barriers to physical activity**

| **Categories and sub-categories of responses generated during NGT sessions** | **Total votes** (across groups)^a^ | **Group types endorsing response**^b^ |
| --- | --- | --- |
| **Facilitators** |  |  |
| Proximity to destinations | 63 | LW/HC, LW/MC, HW/HC |
| Home close to park, walking track, cycling track, swimming pool, gym | 32 |  |
| Community centre, public facilities and shopping within walking distance from home | 17 |  |
| Having park and shopping centre close by | 8 |  |
| Having Chinese club close by | 6 |  |
| Easy access to destinations for physical activity regardless of distance) | 34 | HW/MC, LW/HC |
| Community centre providing physical activity (sports and non-sports) | 18 |  |
| Affordable/cheap centre and facilities for physical activity | 4 |  |
| Easy access to physical activity facilities/community recreation centre for elderly | 12 |  |
| Access to social group/activities | 28 | HW/MC, LW/HC, LW/MC, HW/HC |
| Outdoor physical activity organised by volunteer organisations | 12 |  |
| Having physical activity group close by | 3 |  |
| Having Chinese Seniors Club and University of Third Age (U3A) | 8 |  |
| Diversity in activities on offer on seniors’ day | 5 |  |
| Information available on health and community events, initiatives or services in Chinese | 18 | HW/MC |
| Providing Chinese information about seniors' welfare, health, community services, policy, etc. | 18 |  |
| Providing Chinese books / papers in the library [generated item not scored as important] | 0 |  |
| Home environment providing opportunities for physical activity | 17 | LW/HC, LW/MC, HW/HC |
| Having own garden | 4 |  |
| Need to do house maintenance | 4 |  |
| Having physical activity facilities at home | 9 |  |
| Social support for physical activity | 14 | HW/MC, HW/HC |
| Having a good physical activity instructor | 11 |  |
| Having friends that are physically active | 1 |  |
| Providing Chinese speaking physical activity instructor volunteers at recreation centre | 2 |  |
| Enjoying household physical activity [characteristic describing Chinese older residents in the community] | 14 | HW/HC |
| Chinese older adults enjoying gardening and doing housework | 14 |  |
| Opportunities to facilitate integration in the community | 13 | HW/MC |
| Providing networking opportunities for Chinese and English speaker seniors together in the community | 13 |  |
| Quality public transport | 8 | HW/MC |
| Providing bus stop name/stop, number at each bus stop, and inside the bus as a notification for the next stop | 8 |  |
| Pet ownership | 5 | LW/MC, HW/HC |
| Owning a pet | 5 |  |
| Safety from traffic | 5 | LW/MC |
| The road near house is safe | 5 |  |
| Caring responsibilities | 2 | HW/HC |
| Looking after grandchildren (e.g. taking to/picking up for schools) | 2 |  |
| Living near other Chinese elders | 1 | LW/MC |
| Having Chinese people living close by | 1 |  |
| **Barriers** |  |  |
| Poor/inadequate public transport | 40 | HW/MC, LW/HC, LW/MC, HW/HC |
| Public transport is not convenient (in terms of frequency, regularity and distance) | 38 |  |
| Lack of public transport | 2 |  |
| Language barriers | 36 | HW/MC, LW/HC |
| Inability to communicate with others using English - non-Chinese social environment | 26 |  |
| Language barriers | 10 |  |
| Lack of destinations/facilities supporting physical activity | 34 | LW/HC, LW/MC |
| Not enough activity centres and facilities and not enough activities organised | 1 |  |
| Lack of Chinese community centre where elderly can participate in physical activity | 8 |  |
| Lack of physical activity facilities for elderly in parks | 13 |  |
| Lack of places/destination for elderly to engage in physical activities | 2 |  |
| Destinations close by doesn’t have physical activity facility | 7 |  |
| Park too far away [generated item not scored as important] | 0 |  |
| Walking track lacks chairs for resting | 3 |  |
| Limited social group/activities for Chinese people | 29 | HW/MC, HW/HC |
| Lack of community activities for Chinese people | 2 |  |
| Council taking back Chinese Seniors Club's management rights | 21 |  |
| Chinese Seniors Club stop accepting new members due to issues of the venue, money and time | 6 |  |
| Health-related and socio-economic factors [characteristics describing Chinese older residents in the community] | 28 | HW/MC, HW/HC |
| Lack of money | 12 |  |
| Poor health status | 6 |  |
| Lack of parking discount for seniors | 10 |  |
| Inability to drive [generated item not scored as important] | 0 |  |
| Lack of information on community activities | 23 | HW/MC, LW/HC |
| Lack of information on community activities | 8 |  |
| Lack of Chinese information about activity organised by the community | 15 |  |
| Home environment not conducive to physical activity | 20 | LW/HC, LW/MC |
| Too much housing work therefore lack of time and freedom | 9 |  |
| Own an iPad and watch TV (screen media at home) | 11 |  |
| Lack of social support for physical activity | 9 | LW/HC |
| Lack of support from the family | 9 |  |
| Lack of public housing supporting independence | 3 | HW/MC |
| Lack of public housing therefore unhappy and doesn’t want to go outdoor | 3 |  |

^a^Maximum of 6 votes per participant (3-2-1 scoring). ^b^HW = High Walkability; LW = Low Walkability; MC = Medium % Chinese; HC = High % Chinese.

**Supplementary Table 2: One thing that Chinese older immigrants would change in their own community to help them be more physically active**

| **Categories and sub-categories of responses** | **Total votes** (across groups) | **Group types endorsing response**^a^ |
| --- | --- | --- |
| Access to and improvement of Chinese elderly community centres with facilities for recreational physical activity | 13 | LW/HC, LW/MC, HW/HC, HW/MC |
| Having physical activity opportunities in Chinese elderly community centres | 8 |  |
| Establishing more activity community centres for Chinese elderly | 3 |  |
| Increasing physical activity facilities in community centres | 1 |  |
| Increasing activity diversity in Chinese community centres | 1 |  |
| Improving living arrangements | 12 | HW/MC, LW/HC |
| Living independently (in own home or apartment) | 5 |  |
| Provision of retirement villages or public housing for Chinese elderly allowing independent living | 5 |  |
| Providing home environments that help elderly with mobility problems live independently | 1 |  |
| Improving the home environment so there is more time for leisure | 1 |  |
| Proximity to destinations for recreational physical activity | 5 | HW/MC, LW/MC |
| Improving physical activity facilities in nearby park | 2 |  |
| Having physical activity facilities close by | 1 |  |
| Having Chinese community centres with physical activity facilities close by | 2 |  |
| Reducing language barriers | 4 | HW/MC, LW/HC |
| More information on physical activity opportunities and services in Chinese | 2 |  |
| Enhance ability to communicate in English (e.g., provision of English courses) | 2 |  |
| Improvements in the neighbourhood environment | 1 | HW/MC |
| Improving neighbourhood aesthetics and safety | 1 |  |
| Reducing financial barriers | 1 | HW/HC |
| Having senior discount for parking | 1 |  |
| Opportunities to facilitate integration | 1 | HW/MC |
| Increase networking opportunities for English and Chinese older adults | 1 |  |

^b^HW = High Walkability; LW = Low Walkability; MC = Medium % Chinese; HC = High % Chinese

**Supplementary Table 3: Built and social environmental facilitators and barriers to eating a healthy diet**

| **Categories and sub-categories of responses generated during NGT sessions** | **Total votes** (across groups)^a^ | **Group types endorsing response**^b^ |
| --- | --- | --- |
| **Facilitators**^c^ |  |  |
| High food safety standards/regulations | 59 | HW/MC, LW/HC, LW/MC, HW/HC |
| Having high food safety standards | 41 |  |
| Having strict food regulations (e.g. close monitoring to prevent shop for selling expired food products) | 11 |  |
| Having fish and meat free of contaminants and additives | 7 |  |
| Providing educational information on healthy eating in the community | 44 | HW/MC, LW/HC, LW/MC |
| Promotion and talks on healthy eating in the community in Chinese | 10 |  |
| Chinese newspapers providing healthy diet information | 12 |  |
| Community groups and health services providing talks/workshops on healthy eating in the community | 22 |  |
| Family/household members social support for a healthy diet | 22 | LW/MC, HW/HC |
| Free to choose healthy foods because living alone (without children) | 5 |  |
| Living with people who have knowledge about or follow a healthy diet | 17 |  |
| Availability of healthy foods in grocery stores | 20 | HW/MC, HW/HC, LW/HC |
| Availability of affordable fruits and vegetables | 8 |  |
| Shops selling fresh produce | 10 |  |
| Supermarkets selling healthy foods (e.g., organic food) | 2 |  |
| Food nutritional labelling in Chinese | 13 | LW/MC, LW/HC, HW/HC |
| Food products to have Chinese food labelling to indicate whether the product is a healthy option or not | 13 |  |
| Preference for a healthy diet and healthy cooking practices [characteristic describing Chinese older residents in the community] | 9 | LW/MC, HW/HC |
| Healthy eating and cooking habits (of Chinese elderly in the community) | 9 |  |
| Proximity to Chinese grocery store | 7 | LW/MC |
| Have Asian grocery shop nearby | 7 |  |
| Growing vegetables in the garden | 0 | LW/MC |
| People can grow vegetables in their own garden [generated item not scored as important] | 0 |  |
| **Barriers** |  |  |
| Lack of family/household members social support for a healthy diet | 54 | HW/MC, LW/HC, LW/MC, HW/HC |
| Living with family members who prefer unhealthy food (e.g., pizza and deeply fried food) | 19 |  |
| Living with children who prefer unhealthy food (e.g., deeply fried food) | 35 |  |
| Financial barriers to purchasing healthy foods | 26 | HW/MC, HW/HC |
| Government reducing pension allowance - healthy foods are unaffordable | 10 |  |
| Healthy food products being more expensive than unhealthy food products | 11 |  |
| Eating leftovers, to avoid wasting food and to save money | 5 |  |
| High availability or prevalence of unhealthy food options available in food outlets | 23 | HW/MC, HW/HC |
| Restaurants selling unhealthy foods and adopting unhealthy cooking practices | 10 |  |
| Shops and supermarkets providing lots of foods and beverages high in sugar and fat content | 13 |  |
| Cultural preference for an unhealthy diet and unhealthy cooking practices [characteristic describing Chinese older residents in the community] | 19 | LW/MC, LW/HC, HW/HC |
| Chinese older adults from some parts of China preferring unhealthy foods (e.g., fried foods; pickled foods) | 19 |  |
| Many traditional Chinese festival events provide unhealthy food (e.g. BBQ sausage) [generated item not scored as important] | 0 |  |
| Distance to grocery store | 14 | HW/MC, LW/MC |
| Grocery shops being too far away from home | 14 |  |
| Limited availability of fresh vegetables | 9 | HW/HC |
| Only a few varieties of vegetables being available in grocery shops | 7 |  |
| Foods in supermarkets not being fresh | 2 |  |
| Poor adherence to food safety standards/regulations | 6 | HW/HC |
| Some Chinese groceries try to cover the expire dates of foods | 6 |  |
| Unable to grow fruit and vegetables at home | 4 | HW/MC |
| Feral animals eating home-grown fruit and vegetables | 4 |  |
| Language barriers to reading food nutritional labels | 4 | LW/MC |
| Food labelling not in Chinese | 4 |  |
| Poor public transport (low frequency) | 2 | LW/MC |
| Public transport is inconvenient (low frequency especially during weekends) | 2 |  |
| Misleading or inaccurate educational information on healthy eating | 1 | LW/MC |
| A lot of 'healthy' diet information on products which lack of scientific evidence | 1 |  |

^a^Maximum of 6 votes per participant (3-2-1 scoring). ^b^HW = High Walkability; LW = Low Walkability; MC = Medium % Chinese; HC = High % Chinese. ^c^Additional votes = 12 additional votes for ‘Facilitators’ due to scored items listing two factors being counted twice under different response categories.

**Supplementary Table 4: One thing that Chinese older immigrants would change in their own community to help them follow a healthy diet**

| **Categories and sub-categories of responses** | **Total votes** (across groups) | **Group types endorsing response**^a^ |
| --- | --- | --- |
| Better provision of educational information on healthy eating in the community | 7 | LW/HC, LW/MC, HW/MC |
| Having community groups and health services deliver talks/workshops on healthy eating | 2 |  |
| Information of healthy diets provided at medical check-ups | 1 |  |
| Information on healthy diets provided by local Chinese newspapers | 2 |  |
| Press, internet and TV programs dissemination information on healthy diets | 1 |  |
| Having more opportunities to communicate with Western people about healthy eating | 1 |  |
| Better access to grocery stores and/or fresh produce | 7 | HW/MC, LW/HC, HW/HC |
| More shops (shopping areas, grocery shops) selling vegetables | 3 |  |
| Having a produce marker nearby | 1 |  |
| Having larger amounts and varieties of vegetables/fruit to buy | 3 |  |
| Improve access to and/or management of Chinese grocery stores | 4 | LW/MC, HW/HC |
| Improve the management of Chinese groceries | 3 |  |
| Having at least one Asian grocery store nearby | 1 |  |
| Improved public transport | 4 | HW/MC, LW/MC |
| Improve options, convenience and frequency of public transport | 4 |  |
| Reduce unhealthy food options in food outlets | 2 | LW/HC, HW/HC |
| Reduce the amount of junk food in supermarkets | 1 |  |
| Restaurants replacing fried with steamed food | 1 |  |
| Interventions aimed at improving diet-related habits | 2 | HW/HC |
| Teaching older adults how to change their behaviour to buy healthy foods | 1 |  |
| Support initiatives that promote the adoption of smokeless ovens | 1 |  |
| Financial independence | 1 | HW/MC |
| Financial independence allowing to take care of own grocery shopping | 1 |  |

^b^HW = High Walkability; LW = Low Walkability; MC = Medium % Chinese; HC = High % Chinese

**Supplementary Table 5: Built and social environmental facilitators and barriers to social contacts**

| **Categories and sub-categories of responses generated during NGT sessions** | **Total votes** (across groups)^a^ | **Group types endorsing response**^b^ |
| --- | --- | --- |
| **Facilitators^c^** |  |  |
| Proximity to destinations and activities | 72 | LW/HC, LW/MC, HW/HC, HW/MC |
| Having shops and/or restaurants close by | 11 |  |
| Having a library close by | 14 |  |
| Having places for various activities (e.g.: Art centre, sports centre and community garden) close by | 15 |  |
| Having Chinese GPs close to home | 7 |  |
| Having a Chinese community centre (e.g., Chinese senior club) nearby | 23 |  |
| Having many interesting activities for Chinese elderly close by | 2 |  |
| Availability of community services and media in Chinese | 24 | HW/MC, HW/HC, LW/MC |
| Services in the community provided in both English and Chinese (medical, shops, banks, volunteers and centres) | 17 |  |
| Having access to Chinese newspapers and magazines | 7 |  |
| Opportunities to learn English | 19 | HW/MC, LW/MC |
| Providing English courses in the community | 15 |  |
| Government providing English training classes | 3 |  |
| Translator application on mobile phone (use to communicate with English speakers) | 1 |  |
| Access to destinations and activities | 19 | HW/MC, LW/MC, HW/HC |
| Chinese community and clubs organising many activities in the community | 10 |  |
| Having access to a Chinese library | 2 |  |
| Having access to many shops | 4 |  |
| Having access to Chinese churches | 3 |  |
| Good public transport | 18 | LW/MC, HW/HC |
| Having good public transport | 4 |  |
| Public transport being convenient | 14 |  |
| Living near or with Chinese people | 10 | LW/HC, LW/MC |
| Chinese-speaking people living close by | 7 |  |
| Living with Chinese people | 3 |  |
| Unlimited access to telecommunication | 2 | LW/MC |
| Unlimited time for telephone calls | 2 |  |
| **Barriers** |  |  |
| Poor public transport | 66 | HW/MC, LW/HC, LW/MC, HW/HC |
| Inconvenient public transport | 34 |  |
| Public transport stop signs and bus numbers not clear | 21 |  |
| Insufficient public transport concession fares for older adults | 10 |  |
| Language barriers | 49 | HW/MC, LW/HC, LW/MC, HW/HC |
| People not being able to speak Chinese in the community | 37 |  |
| Not being able to speak English, and others not being able to speak Chinese | 10 |  |
| Magazines about the community all in English | 2 |  |
| Limited or poor access to destinations and social group/activities for Chinese people (or residents) | 41 | HW/HC, HW/MC, LW/MC |
| Limited capacity of Chinese senior clubs | 14 |  |
| Lack of funding for Chinese senior clubs | 13 |  |
| Lack of activity centres and facilities for Chinese elderly nearby | 14 |  |
| Living separately from other Chinese people (public housing arrangements) | 5 | LW/MC |
| Policy for public housing causes Chinese people to live separately | 5 |  |
| Cultural differences limiting integration | 4 | HW/HC |
| Cultural differences between Chinese and English | 4 |  |
| Health-related and personality factors [characteristics describing Chinese older residents in the community] | 3 | HW/MC |
| Introvert personality [characteristics describing Chinese older residents in the community] | 2 |  |
| Poor health status of Chinese elderly living in the community | 1 |  |

^a^Maximum of 6 votes per participant (3-2-1 scoring). ^b^HW = High Walkability; LW = Low Walkability; MC = Medium % Chinese; HC = High % Chinese. ^c^Additional votes = 1 additional votes for ‘Facilitators’ and 6 for ‘Barriers’ due to scored items listing two factors being counted twice under different response categories.

**Supplementary Table 6: One thing that Chinese older immigrants would change in their own community to help them have contacts with people**

| **Categories and sub-categories of responses** | **Total votes** (across groups) | **Group types endorsing response**^a^ |
| --- | --- | --- |
| Improving access to places for social/group activities for Chinese elderly | 7 | LW/HC, HW/MC |
| Establishing or increase the number of activity / community centres for Chinese elderly | 6 |  |
| Providing more parking space at existing Chinese senior clubs | 1 |  |
| Increasing the number of activities in the community for Chinese elderly | 3 | HW/HC |
| Increasing activity diversity in Chinese senior clubs | 1 |  |
| Increasing the number of free activities for Chinese elderly in the community | 2 |  |
| Improving public transport | 7 | HW/MC, LW/HC, LW/MC |
| Improving public transport (access and frequency) | 5 |  |
| Provision of free public transport | 2 |  |
| Provision of opportunities for the improvement of English language skills | 2 | HW/MC, LW/MC |
| Provision of English courses | 1 |  |
| Having networking opportunities with English speakers | 1 |  |
| Provision of services in Chinese | 2 | LW/MC |
| Chinese-speaking police in the community | 1 |  |
| Chinese-speaking staff in hospitals | 1 |  |
| Living near other Chinese people | 2 | LW/MC, HW/MC |
| Local government making it easier for Chinese people to live close together | 1 |  |
| Local government providing public housing for Chinese people | 1 |  |
| Promoting neighbourliness | 1 | LW/HC |
| Having friendly people in the community | 1 |  |
| Housing design to facilitate social contacts | 1 | LW/MC |
| Having a house/apartment with a family room | 1 |  |
| Instrumental support for mobility | 1 | LW/MC |
| Provision of help to Chinese elderly with mobility problems | 1 |  |
| Improved access to telecommunication | 1 | LW/MC |
| Better provision of free wi-fi | 1 |  |

^b^HW = High Walkability; LW = Low Walkability; MC = Medium % Chinese; HC = High % Chinese
